# Eupatorin-Induced Cell Death in Human Leukemia Cells Is Dependent on Caspases and Activates the Mitogen-Activated Protein Kinase Pathway

**DOI:** 10.1371/journal.pone.0112536

**Published:** 2014-11-12

**Authors:** Sara Estévez, María Teresa Marrero, José Quintana, Francisco Estévez

**Affiliations:** Department of Biochemistry and Molecular Biology, University of Las Palmas de Gran Canaria, Las Palmas de Gran Canaria, Spain; Faculty of Medicine & Health Sciences, United Arab Emirates

## Abstract

Eupatorin is a naturally occurring flavone that inhibits cell proliferation in human tumor cells. Here we demonstrate that eupatorin arrests cells at the G_2_-M phase of the cell cycle and induces apoptotic cell death involving activation of multiple caspases, mitochondrial release of cytochrome *c* and poly(ADP-ribose) polymerase cleavage in human leukemia cells. This flavonoid induced the phosphorylation of members of the mitogen-activated protein kinases and cell death was attenuated by inhibition of c-*jun* N-terminal kinases/stress activated protein kinases. Eupatorin-induced cell death is mediated by both the extrinsic and the intrinsic apoptotic pathways and through a mechanism dependent on reactive oxygen species generation.

## Introduction

Flavonoids are polyphenolic compounds that display a vast array of biological activities and they are of great current interest due to their anticancer activities [1]. Some of them induce cell-cycle arrest and apoptosis, which are key features of the action of chemotherapeutic drugs on leukemia cells [2]. Apoptosis can occur with or without the activation of caspases, a family of aspartate-specific cysteine proteases which are generally synthesized as zymogens and activated by proteolytic cleavage [3]. There are two major caspase activation pathways [4]. The extrinsic pathway involves cell surface death receptors, such as tumor necrosis factor, Fas and TRAIL receptors, and is dependent on the initiator caspase-8 which cleaves and activates the downstream effector caspases (caspase-3, -6 and -7), inducing a cascade of caspases. The intrinsic pathway involves the activation of procaspase-9 by cytochrome *c* released from mitochondria, which cleaves and activates downstream effector caspases-3, -6 and -7, which in turn target key structural and regulatory proteins for proteolysis to effect cell death [5]. Both caspase-8 and caspase-9 activate caspase-3 which is responsible for breaking specific cellular proteins during apoptosis [6].

Mitogen-activated protein kinases (MAPKs) are a family of proline-directed serine/threonine protein kinases that control cell proliferation, differentiation and apoptosis. There are three major pathways of MAPKs: the extracellular signal-regulated kinases (ERKs) 1/2, the c-*jun* N-terminal kinases/stress activated protein kinases (JNK/SAPK) and the p38 mitogen-activated protein kinases (p38^MAPK^). ERK 1/2 is predominantly involved in cell growth and survival signals, whereas JNK/SAPK and p38^MAPK^ are activated in response to stress and growth factors and mediate signals that regulate apoptosis [7].

Eupatorin (3′,5-dihydroxy-4′,6,7-trimethoxyflavone) is a flavone which has been previously isolated from several medicinal plants, including *Tanacetum vulgare* [8], *Lantana montevidensis* [9] and *Orthosiphon stamineus* [10], and has been shown to inhibit the proliferation of human and murine cancer cell lines [9]. The anti-proliferative effect of eupatorin against MDA-MB-468 human breast cancer cells has been attributed to cytochromes P450 CYP1 enzymes-mediated metabolism [11]. However, the potential significance of eupatorin in antitumor therapy in leukemia cells is largely unexplored, to date. In the present study we have evaluated its effects on cell cycle and the role of caspases and the MAPK pathway. Furthermore, the formation of reactive oxygen species (ROS) was investigated to address whether they play a pivotal role in the apoptotic effect of eupatorin in leukemia cells.

## Material and Methods

### Reagents

Eupatorin was purchased from Extrasynthese (Genay Cedex, France). The following antibodies were used according to the manufacturer's instructions: poly(ADP-ribose) polymerase (PARP), mouse monoclonal; cytochrome *c*, mouse monoclonal; caspase-7, mouse monoclonal; caspase-8, rabbit polyclonal; Bax, rabbit polyclonal; Bid, rabbit polyclonal; and AIF, rabbit polyclonal (BD PharMingen, San Diego, CA, USA); Smac/DIABLO, mouse monoclonal (BD Transduction Laboratories); caspase-3, rabbit polyclonal (Assay Designs, Ann Arbor, MI, USA); caspase-4, caspase-6 and caspase-9, mouse monoclonal (Medical & Biological Laboratories, Nagoya, Japan); Bcl-2, mouse monoclonal (Santa Cruz Biotechnology, Santa Cruz, CA, USA); β-actin, mouse monoclonal (Sigma, Saint Louis, MO, USA); cytochrome *c* oxidase (Cox IV), mouse monoclonal (Abcam, Cambridge, UK); JNK/SAPK, Phospho-JNK/SAPK (phosphor T183 + Y185), p44/42 MAP Kinase, Phospho-p44/42 MAP Kinase (T202/Y204), p38^MAPK^ and Phospho- p38^MAPK^ (T180/Y182), rabbit polyclonal (New England BioLabs, Cell Signaling Technology, Beverly, MA, USA). Polyvinylidene-difluoride membranes were purchased from Millipore (Billerica, MA, USA). Secondary antibodies were from GE Healthcare Bio-Sciences AB (Little Chalfont, UK). All other chemicals were obtained from Sigma (Saint Louis, MO, USA).

### Cell culture and Cytotoxicity Assays

HL-60, U937 and Molt-3 cells were obtained from the German Collection of Microorganisms and Cell Cultures (Braunschweig, Germany) and grown in RPMI 1640 medium supplemented with 10% (v/v) heat-inactivated fetal bovine serum, 100 units/ml penicillin and 100 µg/ml streptomycin. The cytotoxicity of eupatorin was analyzed by colorimetric 3-(4,5-dimethylthiazol-2-yl)-2,5-diphenyltetrazolium bromide (MTT) assay as previously described [12] and the concentration required to reduce cell viability by 50% (IC_50_) was determined graphically using the curve fitting algorithm of the computer software Prism 4.0 (GraphPad). Values are means ± SEs from three independent experiments, each performed in triplicate.

### Evaluation of Apoptosis

Fluorescent microscopy, flow cytometric analysis of propidium iodide-stained nuclei and DNA fragmentation assay were performed as described [13]. Apoptosis was also determined by translocation of phosphatidylserine to the cell surface using the annexin V-fluoresceine isothiocyanate (FITC) apoptosis detection kit (BD Pharmingen) according to the manufacturer's protocol.

### Western Blot Analysis

Immunoblot analysis of Bcl-2 family members, caspases, cytochrome *c*, MAPKs and PARP was performed as previously described [13]. Purity of mitochondrial and cytosolic fractions was determined by probing with antibodies to cytochrome *c* oxidase and β-actin, respectively.

### Assay of Caspase Activity

Caspase activity was evaluated by measuring proteolytic cleavage of the chromogenic substrates LEHD-*p*NA (for caspase-9 activity), IETD-*p*NA (for caspase-8 activity), VEID-*p*NA (for caspase-6 activity) and DEVD-*p*NA (for caspase-3 like protease activity) as described previously [12].

### Intracellular Reactive Oxygen Species (ROS) Determination

The generation of peroxides and superoxide was monitored by flow cytometry with the probes 2′,7′-dichlorodihydrofluorescein diacetate (H_2_-DCF-DA) and dihydroethidium (DHE), respectively [14]-[15]. H_2_-DCF-DA is incorporated into cells and deacetylated by intracellular esterases to yield the nonfluorescent 2′,7′-dichlorodihydrofluorescein (H_2_-DCF). H_2_-DCF and DHE are oxidized by H_2_O_2_ (and other peroxides) and superoxide anions, respectively, to yield the highly fluorescent compounds 2′,7′-dichlorofluorescein (DCF) and ethidium. The intensity of fluorescence of DCF and ethidium is proportional to the amount of peroxide and superoxide, respectively, produced by cells. Cells were treated with or without eupatorin and H_2_-DCF-DA (2 µM) or DHE (2 µM) was added to the medium 30 min before the end of incubation with eupatorin. Cells were irradiated with an argon laser at 488 nm, and fluorescence was detected at 525 nm (DCF) and 568 nm (DHE) in the flow cytometer.

### Statistical Analysis

Statistical significance of differences between means of control and treated samples was assessed using Student's *t*-test. *P* values of <0.05 were considered significant.

## Results

### Eupatorin Inhibits the Growth and Cell Viability and Induces Apoptotic Cell Death in Human Leukemia Cell Lines

**Figure 1:**
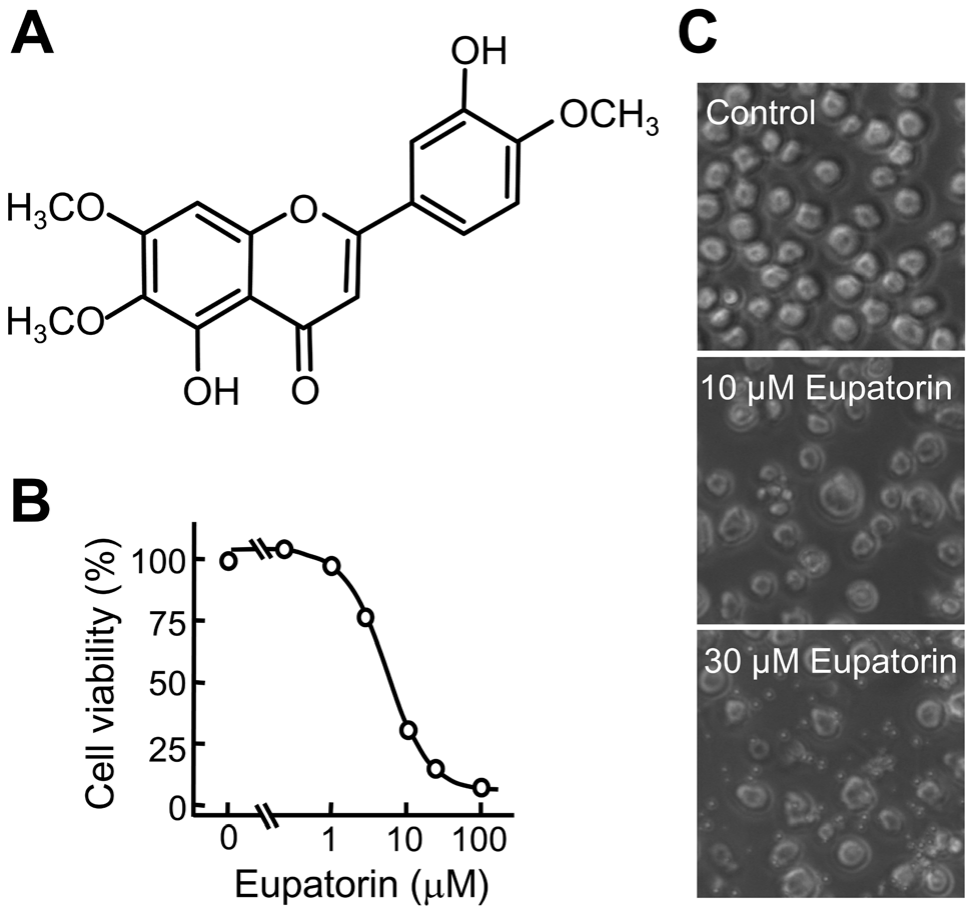
Chemical structure of eupatorin and its effect on human HL-60 cell viability. (A) Structure of eupatorin. (B) Changes in cell viability as determined by the MTT assay. HL-60 cells were cultured in the presence of the indicated concentrations for 72 h, and the results are representative of those obtained in at least three independent experiments. (C) Cells were incubated with vehicle (control) or the indicated concentrations of eupatorin for 24 h and images were obtained with an inverted phase-contrast microscope.

In the present study, we examined the effect of eupatorin (Figure 1A) on the growth of three human leukemia cells and found that human myeloid (HL-60 and U937) and lymphoid (Molt-3) cell lines were highly sensitive to the anti-proliferative effect of this flavonoid. Treatment with eupatorin resulted in a concentration-dependent inhibition of cell viability, with no significant differences among the three cell lines with IC_50_ values of ∼5 µM (Figure 1B). Eupatorin also induced significant morphological changes and an important reduction in the number of cells (Figure 1C).

### Eupatorin Induces Apoptosis in Human Leukemia Cells

**Figure 2:**
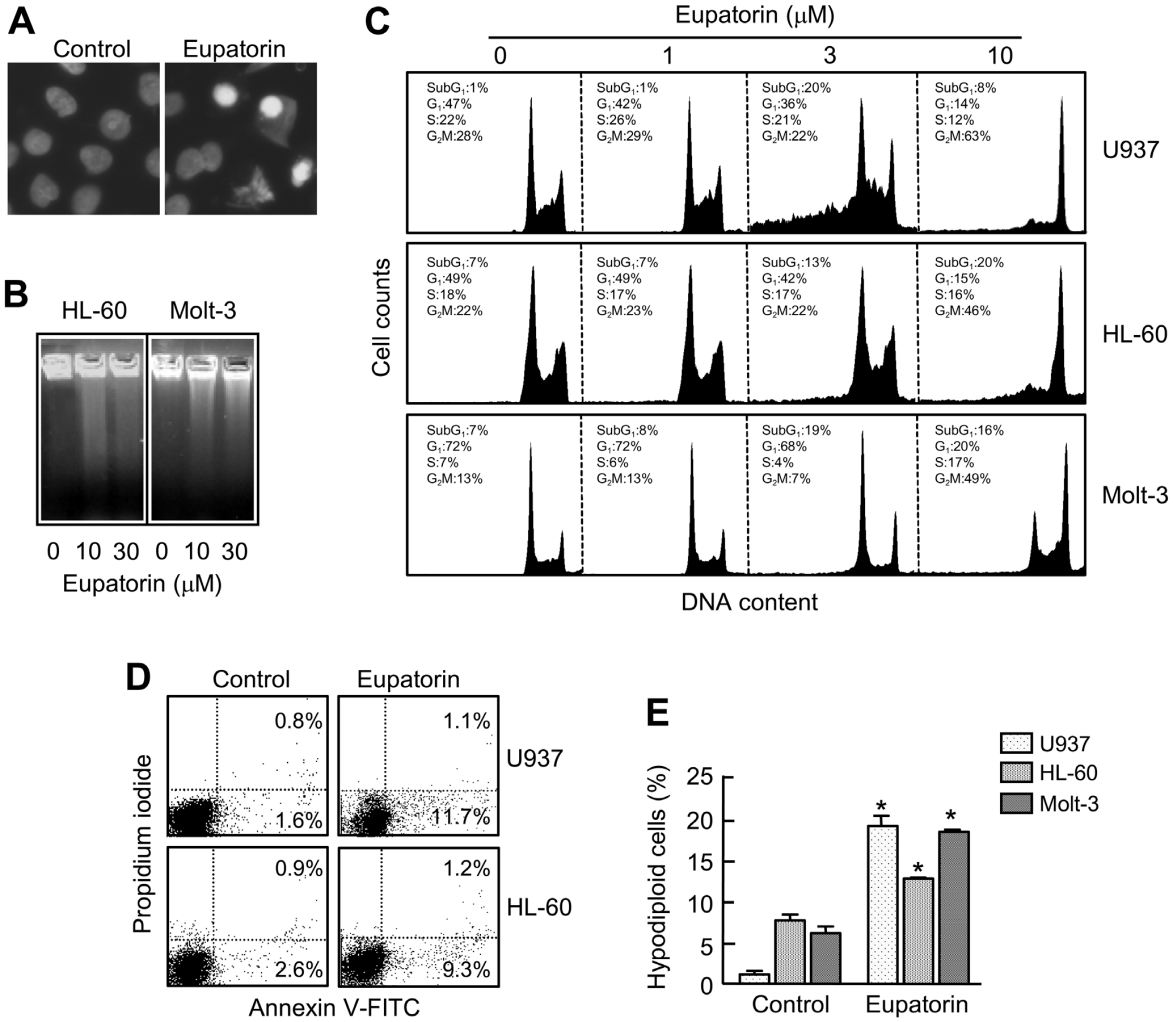
Eupatorin induces apoptosis in human leukemia cells. (A) Fluorescence microscope images of HL-60 cells treated with 3 µM eupatorin or vehicle (control) for 24 h after nuclear staining with bisbenzimide trihydrochloride to evaluate nuclear chromatin condensation. (B) Effect of eupatorin on DNA fragmentation in human tumoral cells. HL-60 and Molt-3 cells were incubated with the indicated concentrations of eupatorin for 24 h and genomic DNA was extracted, separated on an agarose gel and stained with ethidium bromide. (C) Cells were incubated in the presence of the indicated concentrations of eupatorin and the cell cycle phase distribution was determined by flow cytometry. Data originate from three separate experiments. (D) Detection of apoptotic cells by annexin V-FITC and propidium iodide-doubled staining after treatment with 3 µM eupatorin for 24 h. Cells appearing in the lower right quadrant show positive annexin V-FITC staining, which indicates phosphatidylserine switch to the outer leaflet of the plasma membrane, and negative propidium iodide staining, which demonstrates intact cell membranes, both features of early apoptosis. Cells in the top right quadrant are double positive for annexin V-FITC and propidium iodide and are undergoing necrosis. Data originate from three separate experiments. (E) Cells were incubated with 3 µM eupatorin for 24 h and percentage of cells in the sub-G_1_ region was determined by flow cytometry. Error bars represent means±SEs of three independent experiments each performed in triplicate. * indicates *P*<0.05 for comparison with untreated control.

To study the mechanism involved in eupatorin-induced cytotoxicity, we analyzed the nuclei of treated cells using fluorescent microscopy and observed the typical morphologic characteristics of apoptotic cells such as nuclear condensation and fragmented chromatin (Figure 2A). Agarose gel electrophoresis showed that incubation with eupatorin induced DNA fragmentation that is characteristic of apoptotic cells (Figure 2B). To determine whether the decrease of cell growth is mediated via alterations in cell cycle progression, cell cycle phase distribution was evaluated by flow cytometry. As shown in Figure 2C and Table 1, eupatorin (3 µM) caused an accumulation of cells at the G_2_-M phase at the expense of G_1_ phase cell population starting at 6 h of treatment in HL-60, U937 and Molt-3 cell lines. The G_2_-M arrest was higher in U937 cells than in the other cell lines, and this effect was sustained until 12 h in HL-60 and U937 but not in Molt-3. The percentage of sub-G_1_ cells increased after 12 h in the three cell lines. Treatment with this flavonoid also led to the translocation of phosphatidylserine on the outside of the plasma membrane in HL-60 and U937 cells as detected by annexin V-FITC staining (Figure 2D). The percentage of apoptotic cells corresponding to the sub-diploid population increased ∼20-fold, ∼2-fold and ∼3-fold in U937, HL-60 and Molt-3 cells, respectively, after 24 h exposure at a low concentration of eupatorin (Figure 2E).

**Table 1:** Effect of different durations of treatment with eupatorin on cell cycle phase distribution of human leukemia cells.

		% Sub-G_1_	%G_1_	%S	G_2_-M
U937					
6 h	Control	0.9±0.1	45.2±1.1*	22.9±0.4	30.1±1.3*
	Eupatorin	1.5±0.1	18.5±1.4*	26.4±0.3	52.7±1.3*
12 h	Control	0.5±0.1	45.8±0.8*	26.3±0.5*	26.1±0.4*
	Eupatorin	10.5±1.0*	19.0±2.0*	17.4±0.7*	51.6±1.1*
24 h	Control	1.2±0.4	46.9±0.5*	22.2±0.4*	28.2±0.3*
	Eupatorin	19.4±1.3*	35.7±1.7*	20.6±0.5*	21.2±1.0*
HL60					
6 h	Control	6.7±0.3	44.5±0.7*	15.0±0.5	29.1±0.4*
	Eupatorin	8.6±0.5	32.3±0.9*	16.0±0.2	36.7±1.2*
12 h	Control	5.1±0.6	45.5±0.4*	18.0±0.3	27.7±0.2*
	Eupatorin	12.8±0.4*	32.2±1.1*	11.6±0.3	38.4±1.4*
24 h	Control	7.8±0.7	49.7±0.3*	17.9±0.2*	21.8±0.8
	Eupatorin	12.9±0.1*	42.2±0.4*	17.4±0.5*	21.9±0.8
Molt-3					
6 h	Control	6.2±0.8	70.2±1.2	7.6±0.4	16.2±0.4
	Eupatorin	5.1±0.3	64.9±0.4*	9.6±0.3	21.1±0.4*
12 h	Control	9.1±1.4	70.7±1.7	7.2±0.3	7.2±0.2
	Eupatorin	13.3±1.7*	64.8±3.5	5.2±0.6	6.0±0.6
24 h	Control	7.1±0.2	70.2±0.5	6.5±0.3	13.4±0.2*
	Eupatorin	18.6±1.8*	67.9±1.7	4.0±0.2	7.2±0.4

Cells were cultured with 3 µM eupatorin for the indicated period of times and the cell cycle phase distribution was determined by flow cytometry. The values are means ± SE of three independent experiments with three determinations in each. Asterisks indicate a significant difference (*P*<0.05) compared with the corresponding controls.

### Eupatorin-induced Cell Death is Dependent on Caspases

**Figure 3:**
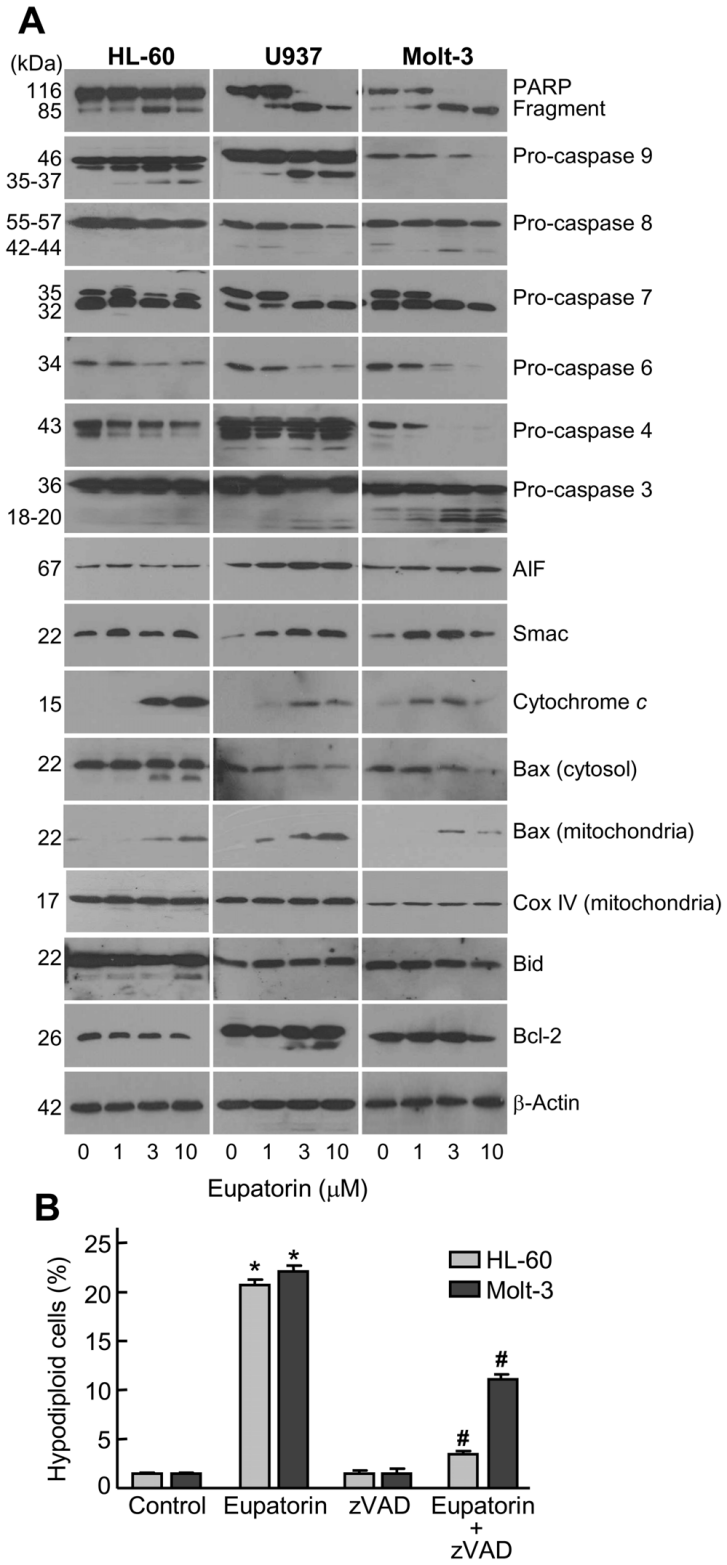
Involvement of caspases in the apoptosis induced by eupatorin on human leukemia cells. (A) The cells were incubated in the presence of the indicated concentrations of eupatorin and cell lysates (or cytosolic extracts in the case of cytochrome *c*, AIF and Smac) were assayed by immunoblotting. β-Actin and Cox IV (cytochrome *c* oxidase) were used as loading controls in cytosol and mitochondria, respectively. (B) Effect of cell-permeable pan-caspase inhibitor z-VAD-fmk on eupatorin-stimulated apoptosis. HL-60 and Molt-3 cells were incubated with 10 µM eupatorin for 24 h, in absence or presence of z-VAD-fmk (100 µM) and apoptotic cells (i.e. hypodiploid DNA content) were quantified by flow cytometry after staining with propidium iodide. * indicates *P*<0.05 for comparison with untreated control, # indicates *P*<0.05 for comparison with eupatorin treatment alone.

To explore the role of caspases, cells were treated with increasing concentrations of eupatorin for 24 h and caspases were determined by Western blot. The results indicate that this compound activates the cleavage of inactive procaspases-9 and -8 and the proteolytic processing of executioner procaspases-7, -6 and -3 (Figure 3A). Poly(ADP-ribose)polymerase (PARP) protein, one of the substrates of caspase-3, was cleaved into the 85 kDa fragment in cells exposed to eupatorin. We also observed a decrease of pro-caspase-4, suggesting that eupatorin induces cleavage of this zymogen which is involved in endoplasmic reticulum stress [16]. To know whether eupatorin-induced apoptosis is mediated through mitochondrial cytochrome *c* release, cytosolic preparations were analyzed and a significant rise in the levels of cytochrome *c* in the cytosol was observed after treatment with 1 µM (Molt-3) or 3 µM (HL-60 and U937) eupatorin. Moreover, eupatorin also induced the release of other mitochondrial proteins such as apoptosis-inducing factor (AIF) and Smac/DIABLO to the cytosol.

In light of mitochondria are important in programmed cell death signaling pathways and since this cell death pathway is regulated by the Bcl-2 family proteins, we next investigated whether changes in the expression or in the localization of these proteins were associated with the cell death. Treatment of HL-60 and Molt-3 cells with eupatorin at levels sufficient to induce apoptosis decreased the expression of the Bcl-2 protein, whereas in U937 cells 3 µM eupatorin induced Bcl-2 cleavage. However, Bax was found predominantly in the cytosolic fraction in untreated cells and incubation with eupatorin induced a redistribution of this apoptotic factor from cytosolic to the mitochondrial compartment. Interestingly, a 18 kDa cleavage fragment generated from the full length 22 kDa Bax was only detected in HL-60 cells treated with the flavonoid. To explore whether eupatorin leads to the activation of the BH3-only protein that mediates caspase-8-induced cytochrome *c* release from mitochondria in response to activation of cell surface death receptors, the levels and/or the cleavage of Bid was also analyzed. A decrease in Bid protein levels was observed in HL-60 and Molt-3 cells, presumably reflecting a cleavage or activation while no significant changes was detected in U937 cells. Although there was not a clear Bid cleavage in U937 cells pro-caspase-8 was significantly reduced as revealed by the decrease in the amount of the 55-57-kDa pro-enzyme. To confirm that eupatorin-triggered cell death requires the activation of caspases, HL-60 and Molt-3 cells were exposed to eupatorin in the presence or absence of the pan-caspase inhibitor z-VAD-fmk, after which the extent of apoptosis was examined. The results (Figure 3B) show a significant reduction in hypodiploid cells in the presence of the general caspase inhibitor, which suggests that eupatorin induces cytotoxicity by a caspase-dependent mechanism.

**Figure 4:**
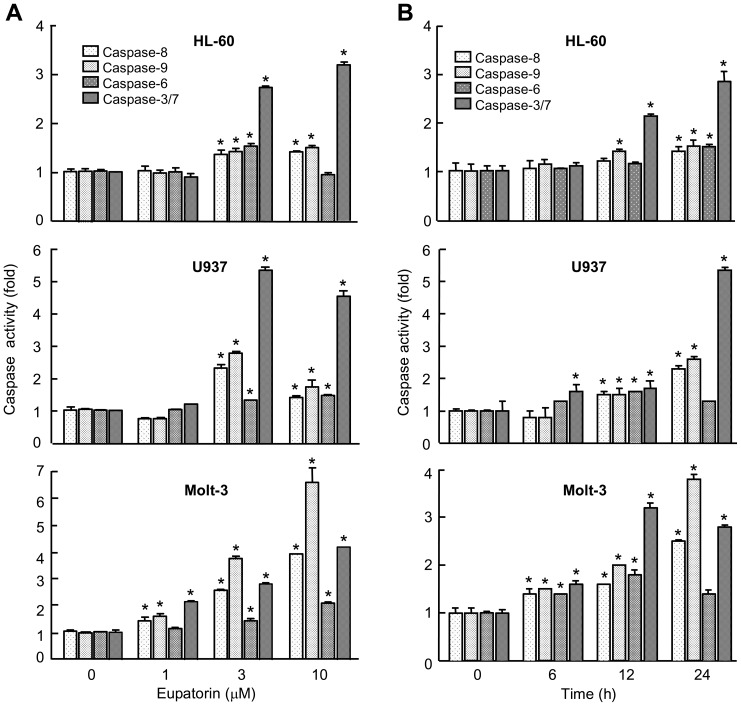
Dose-dependence and kinetics of caspase activation in response to eupatorin in human leukemia cells. (A) Caspase activation in response to eupatorin. Cells were treated with the indicated concentration of eupatorin and harvested at 24 h. Cell lysates were assayed for caspase-9, caspase-8, caspase-6 and caspase-3/7 activities using the LEHD-*p*NA, IETD-*p*NA, VEID-*p*NA and DEVD-*p*NA colorimetric substrates, respectively. The values represent fold induction of caspase activity relative to untreated control. (B) Kinetics of caspase activation in response to eupatorin. Cells were treated with 3 µM eupatorin, harvested at the indicated times, and cell lysates were assayed and the results are expressed as above. * indicates *P*<0.05 for comparison with untreated control.

As processing of caspases is not always correlated with activity, enzymatic activities were also investigated. As shown (Figure 4A), induction of caspase-3/7, caspase-8 and caspase-9 activities increased in presence of eupatorin after 24 h of treatment and this effect was dose-dependent. To study the time-course of caspase activation, cells were treated with this flavone and then harvested at various intervals. Induction of both initiator caspase-9 and -8 activities was significantly detectable at 6 h (Molt-3) or at 12 h (HL-60 and U937) of treatment (Figure 4B). These findings indicated that eupatorin induces simultaneous caspase-9 and -8 activation and also promotes a marked activation of caspases -3/7, whereas the executioner caspase-6 showed a lesser degree of activation.

### Eupatorin Activates MAPKs

**Figure 5:**
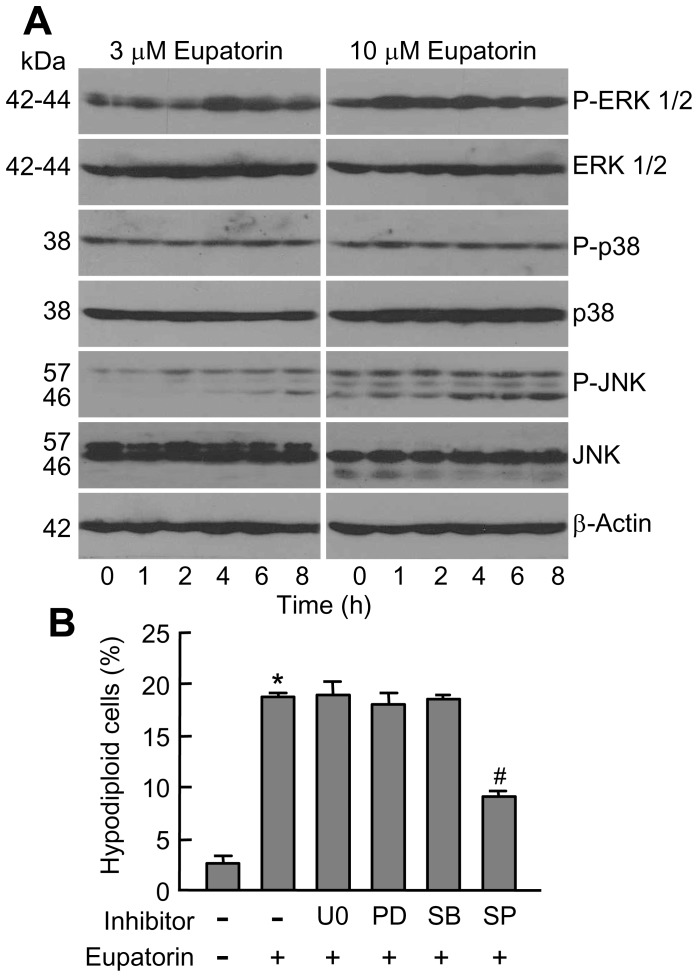
Eupatorin induces phosphorylation of MAPKs. (A) Time- and concentration-dependent phosphorylation of MAPKs by eupatorin in HL-60 cells. Representative blots are shown. (B) Cells were pretreated with U0126 (U0, 10 µM), PD98059 (PD, 10 µM), SB203580 (SB, 2 µM) and SP600125 (SP, 10 µM) for 1 h and then incubated with eupatorin for 24 h and hypodiploid cells were quantified by flow cytometry. * indicates *P*<0.05 for comparison with untreated control, # indicates *P*<0.05 for comparison with eupatorin treatment alone.

Since the MAPKs play an essential role in cell growth and apoptosis, the effects of eupatorin on the activation of these protein kinases were investigated in HL-60 cells. The results show a rapid phosphorylation (1-2 h) of phospho-ERK 1/2 and phospho-JNK/SAPK which remained increased for at least 8 h while the activation of p38^MAPK^ was not detected (Figure 5A). To determine the possible involvement of MAPKs in eupatorin-induced apoptosis, we investigated the effects of specific inhibitors against mitogen-activated extracellular kinases 1/2 (MEK1/2) (PD98059 and U0126), p38^MAPK^ (SB203580) and JNK/SAPK (SP600125). Neither the inhibition of mitogen-activated extracellular kinases 1/2 (MEK1/2) nor the inhibition of the p38^MAPK^ affected eupatorin-induced cell death. However, treatment of cells with the JNK/SAPK inhibitor significantly reduced eupatorin-mediated apoptosis (Figure 5B).

### Reactive Oxygen Species (ROS) Are Involved in Eupatorin-induced Cell Death

**Figure 6:**
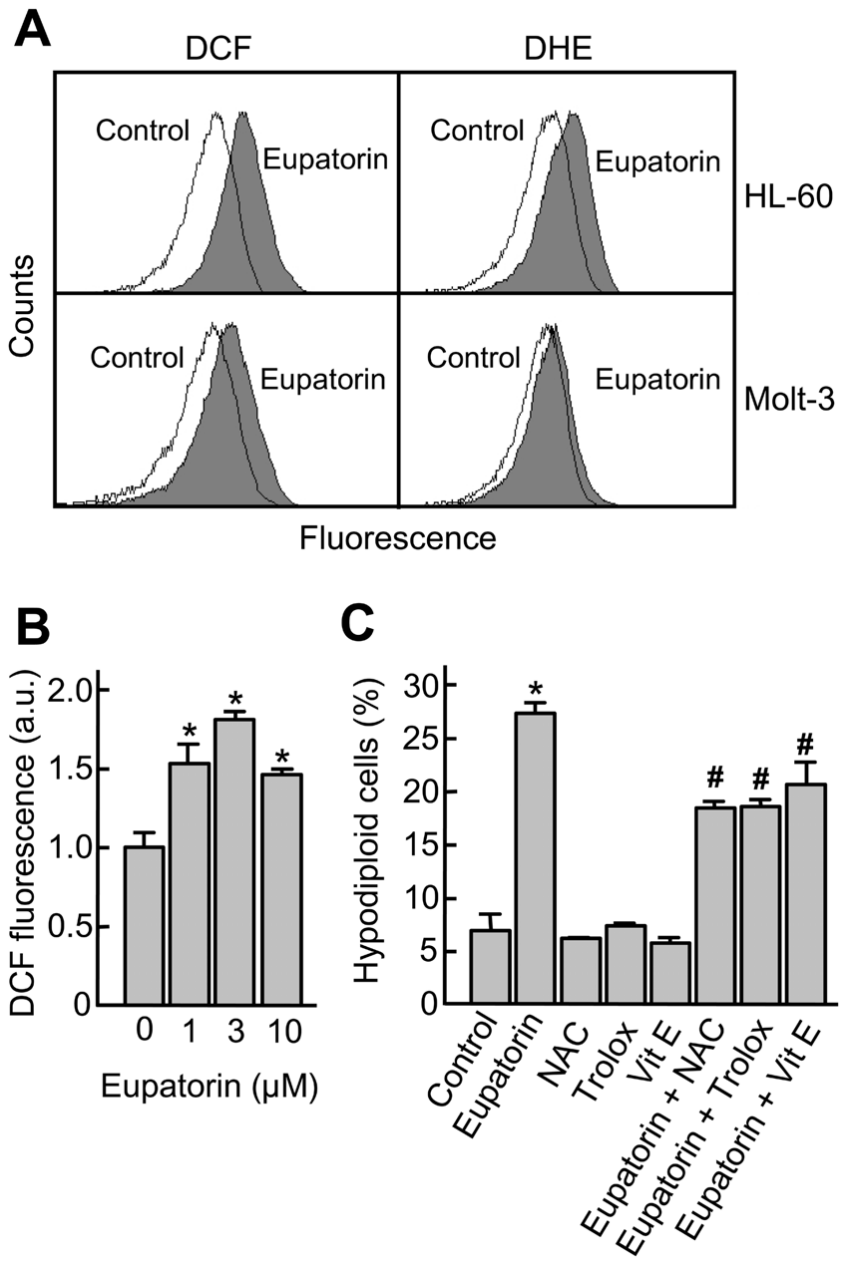
Eupatorin induces ROS generation and scavengers of ROS inhibit cell death. (A) HL-60 and Molt-3 cells were incubated with 10 µM eupatorin for 24 h and 6 h, respectively, and the fluorescence of oxidized H_2_-DCF or DHE was determined by flow cytometry. (B) Molt-3 cells were incubated with eupatorin for 6 h and the H_2_-DCF-derived fluorescence was measured as above. (C) Molt-3 cells were preincubated with *N*-acetyl-l-cysteine (NAC, 10 mM), trolox (2 mM) and vitamin E (25 µM) for 1 h and then incubated with eupatorin (10 µM) for 24 h and apoptotic cells were quantified by flow cytometry. Values represent means ± SE of two independent experiments, each performed in triplicate. * indicates *P*<0.05 for comparison with untreated control, # indicates *P*<0.05 for comparison with eupatorin treatment alone.

Reactive oxygen species are considered to be key mediators of apoptotic signaling and increases in intracellular concentrations may lead to the activation of mitogen-activated protein kinases and cell death. To verify whether ROS are involved in eupatorin-induced apoptosis, cells were stained with the probes 2′,7′-dichlorodihydrofluorescein diacetate (H_2_-DCFDA) and dihydroethidium (DHE) and then analyzed by flow cytometry. As shown in Figure 6A and B, treatment with eupatorin increased the H_2_-DCFDA-derived fluorescence in HL-60 and Molt-3 cells, as indicated by a rightward shift in fluorescence upon flow cytometry, suggesting that the generation of H_2_O_2_ and other peroxides was affected by eupatorin in both cell lines. There was a 2.4-fold and a 1.8-fold increase in the H_2_-DCFDA-derived fluorescence in HL-60 and Molt-3 cells, respectively.

To detect superoxide anion, cells were incubated with DHE, which is relatively specific to superoxide anion and reacts only minimally to hydrogen peroxide. Eupatorin increased the DHE-derived fluorescence (approximately 2-fold) in HL-60 cells. In contrast, we were unable to detect any increase in DHE-derived fluorescence in Molt-3 cells treated with eupatorin.

To investigate whether ROS production is required for eupatorin-induced cell death, Molt-3 cells were preincubated with 10 mM *N*-acetyl-l-cysteine (NAC), 25 µM α-tocopherol (vitamin E) and 2 mM trolox, the hydrosoluble derivative of vitamin E. The results demonstrate that these antioxidants block significantly apoptosis, indicating that ROS generation plays an important role in the mechanism of cell death triggered by eupatorin (Figure 6C).

## Discussion

Although there are efficient treatments for chronic myelogenous leukemia and for acute lymphocytic leukemia, more effective treatments are needed for other forms of acute leukemia. This objective has led to an increasing interest in the research and development of naturally occurring compounds for chemoprevention or recurrence of cancers. In recent years, flavonoids have aroused intense interest because they display a vast array of biological activities, including antitumor properties [17]. In the present study, we demonstrate that eupatorin displays an anti-proliferative effect in three human leukemia cell lines, with similar IC_50_ values. Although this compound promotes condensation and fragmentation of chromatin, there was no clear DNA laddering. This result suggests that other factors may be involved, such as the mitochondrial flavoprotein apoptosis-inducing factor, AIF, which is a cell death-signaling molecule that translocates to the nucleus to induce large-scale DNA fragmentation without DNA laddering, chromatin condensation and cell death [18]. Cell cycle phase distribution analysis indicated that eupatorin causes an accumulation of cells at G_2_-M phase and increases the percentage of cells in the sub-G_1_ region which is considered the fraction of apoptotic cells. Moreover cell death was accompanied by phosphatidylserine externalization.

Many anticancer drugs have been shown to cause the death of sensitive cells by inducing apoptosis. This kind of cell death is considered an important response to most chemotherapeutic drugs in leukemia cells [19]–[21] and can occur with or without the activation of caspases. The results shown here demonstrate that the antiproliferative effect of eupatorin is dependent on caspase activation since apoptosis was completely (HL-60) or partially (Molt-3) inhibited by the pan-caspase inhibitor z-VAD-fmk. These results suggest the involvement of additional pathways of cell death, such as the lysosomal pathway which involves a partial rupture of lysosome membrane and subsequent release of cathepsins into the cytosol [22]. Further studies are required to determine whether lysosomal cathepsins might be also involved in eupatorin-induced cell death.

In many apoptotic responses, mitochondria play a major role in coordinating caspase activation through the release of apoptogenic factors, such as cytochrome *c*, Smac/DIABLO and AIF. Eupatorin induces the release of cytochrome *c* from the intermembrane space, and thus the activation of caspase-9 and caspase-3, indicating that the intrinsic apoptotic pathway plays an important role in the cell death. The presence of cytochrome *c* in the cytosol is essential for the formation and activation of the apoptosome. We also observed the release of other mitochondrial proteins such as Smac/DIABLO and AIF after eupatorin treatment. Smac/DIABLO is a mitochondrial proapoptotic factor which also participates together with cytochrome *c* in the activation of this cascade, while AIF translocates to the nucleus where it contributes to chromatin condensation and large-scale DNA fragmentation [23]. These results suggest that the intrinsic pathway triggered by eupatorin may also operate via caspase-independent mechanisms. However, our findings demonstrate activation and proteolytic processing of the initiator pro-caspase-8, which suggests that this cysteine protease may be important for eupatorin-induced cytotoxicity in leukemia cells. This result is interesting because caspase-8 causes direct activation of executioner caspases and triggers apoptosis despite the mitochondrial protection conferred by the anti-apoptotic members of the Bcl-2 family. Increases in the expression of the anti-apoptotic protein Bcl-2 are associated with cellular resistance to conventional chemotherapeutic drugs, especially in the case of hematologic malignancies [24]. We found that eupatorin treatment decreased the levels of Bcl-2 protein in HL-60 and Molt-3 cells and induced a dose-dependent Bcl-2 cleavage in U937 cells. Previous studies have shown that the anti-apoptotic protein Bcl-2 is converted into a potent pro-apoptotic factor and may enhance cell death by amplifying the caspase cascade and that the cleavage of Bcl-2 by caspase-3 into a 22-kDa fragment induces mitochondrial cytochrome *c* release [25]. Moreover, eupatorin also induced Bax cleavage in HL-60 cells to generate a 18 kDa fragment known to show greater efficiency than the full-length protein in promoting mitochondrial outer membrane permeabilization [26].

Active caspase-8 causes Bid cleavage and translocation of truncated Bid to mitochondria, resulting in mitochondrial permeabilization, which leads to release of mitochondrial proapoptotic factors [27]. Here we found that eupatorin induced a decrease (Molt-3) or a cleavage (HL-60) of Bid protein. Although in most cases truncated Bid seems to be required for inducing mitochondrial outer membrane permeabilization, a possible pro-apoptotic role for full-length Bid has also been suggested [28]–[29]. Eupatorin also stimulated the proteolytic processing and the activation of other executioner caspases, such as caspase-6 and caspase-7, which could cooperate with caspase-3 to induce cell death. Moreover, the endoplasmic reticulum stress signaling pathway might be involved since eupatorin also induced pro-caspase-4 cleavage.

Mitogen-activated protein kinases (MAPKs) regulate cellular proliferation, differentiation and programmed cell death. Two members of this family, c-*jun* N-terminal kinases/stress-activated protein kinases (JNK/SAPK) and p38^MAPK^ play an essential role in triggering cell death in response to various stressors including oxidative stress. In the present study, we show that JNK/SAPK phosphorylation occurred after 1–2 h of treatment with eupatorin and remained elevated for up to at least 8 h and that the inhibition of this kinase by SP600125 significantly decreases eupatorin-induced cell death. These results suggest that JNK/SAPK is required at least in part, for cell death. Eupatorin also stimulated the activation of the MEK/ERK 1/2 pathway but the combination of this flavonoid and the specific mitogen-activated extracellular kinases (MEK) 1/2 inhibitors PD98059 and U0126 did not attenuate nor enhance either cell death. The p38^MAPK^ signaling does not appear to be required for eupatorin-induced apoptosis since there was no clear activation/phosphorylation of p38^MAPK^ and also the p38^MAPK^ inhibitor SB203580 did not attenuate apoptosis.

The reactive oxygen species (ROS) have been implicated as second messengers in several signaling pathways including apoptotic and necrotic cell death [30]. The antiproliferative effect of eupatorin is associated with an increase in the intracellular concentration of ROS, which appear to play a crucial role in the apoptotic process since different antioxidants partially blocked the cell death.

## Conclusions

In summary, the findings of the present study indicate that eupatorin induces cell death in human leukemia cell lines at concentrations which might be achieved *in vivo*. Eupatorin induces cytotoxicity via G_2_-M phase cell-cycle arrest and apoptosis through a caspase-dependent mechanism, is associated with cytochrome *c* release, and is dependent on ROS generation. Eupatorin induces the activation of the MAPK pathway and activation of JNK/SAPK is essential for cell death. These results provide a basis to further evaluate the potential applications of eupatorin and/or structurally similar compounds in the fight against cancer.
